# Optimization of microbubble enhancement of hyperthermia for cancer therapy in an *in vivo* breast tumour model

**DOI:** 10.1371/journal.pone.0237372

**Published:** 2020-08-14

**Authors:** Deepa Sharma, Holliday Cartar, Niki Law, Anoja Giles, Golnaz Farhat, Michael Oelze, Gregory J. Czarnota

**Affiliations:** 1 Imaging Research and Physical Sciences, Sunnybrook Health Sciences Centre, Toronto, Ontario, Canada; 2 Department of Radiation Oncology, Sunnybrook Health Sciences Centre, Toronto, Ontario, Canada; 3 Departments of Medical Biophysics, and Radiation Oncology, Faculty of Medicine, University of Toronto, Toronto, Ontario, Canada; 4 Department of Electrical and Computer Engineering, Beckman Institute, University of Chicago Illinois at Urbana Champaign, Illinois, United States of America; California Pacific Medical Center, UNITED STATES

## Abstract

We have demonstrated that exposing human breast tumour xenografts to ultrasound-stimulated microbubbles enhances tumour cell death and vascular disruption resulting from hyperthermia treatment. The aim of this study was to investigate the effect of varying the hyperthermia and ultrasound-stimulated microbubbles treatment parameters in order to optimize treatment bioeffects. Human breast cancer (MDA-MB-231) tumour xenografts in severe combined immunodeficiency (SCID) mice were exposed to varying microbubble concentrations (0%, 0.1%, 1% or 3% v/v) and ultrasound sonication durations (0, 1, 3 or 5 min) at 570 kPa peak negative pressure and central frequency of 500 kHz. Five hours later, tumours were immersed in a 43°C water bath for varying hyperthermia treatment durations (0, 10, 20, 30, 40, 50 or 60 minutes). Results indicated a significant increase in tumour cell death reaching 64 ± 5% with combined treatment compared to 11 ± 3% and 26 ± 5% for untreated and USMB-only treated tumours, respectively. A similar but opposite trend was observed in the vascular density of the tumours receiving the combined treatment. Optimal treatment parameters were found to consist of 40 minutes of heat with low power ultrasound treatment microbubble parameters of 1 minute of sonification and a 1% microbubble concentration.

## Introduction

Hyperthermia (HT) therapy, defined as the moderate elevation of body tissues to temperatures of 39–45°C, has been used as a compelling treatment of cancers for centuries [1]. The hindrance to its adoption into regular clinical use has come from the lack of available technologies delivering effective and homogenous heat to target sites, as well as limitations in temperature monitoring techniques. More recently, however, with the development of improved techniques, there has been a renewed interest in HT in oncology. HT is a valuable therapy because of its inherent tumour selective effect. At temperatures between 40–44°C, tumour cells *in vivo* are irreversibly damaged resulting in cell death while normal cells are seemingly spared [2]. Most normal tissues can withstand exposure to temperatures up to 44°C for 1 hour without permanent damage [2, 3]. Tumour sensitivity to HT is not well understood but can be largely attributed to the abnormal physiology found in solid tumours. The chaotic vasculature found in tumours often leads to areas of hypoxia and low pH, factors which render cells more thermosensitive [2]. Tumour sensitivity can also vary depending on the cell cycle phase (dividing cells in M and S-phase are more susceptible to heat-induced damage) [4] and maybe influenced by the differential expression of heat shock proteins on tumour cell membranes [2, 5, 6]. HT has traditionally been most effective in cancer therapy as a sensitizing agent to other primary cancer therapies. The clinical use of HT to induce radiosensitization (mainly through inhibition of DNA repair) and chemosensitization (through increased drug uptake, higher intra-tumour drug concentrations due to increased blood flow, and enhanced drug toxicity) is well documented [2, 7, 8]. As well, its effectiveness in treating a wide variety of tumour types has been demonstrated clinically and includes head and neck, breast, brain, bladder, cervix, rectum, lung, esophagus, vulva and vagina, non-melanoma and melanoma skin cancer, and sarcomas [7]. The mechanisms for heat-induced cytotoxicity are thought to be vast and complex. However, the critical event appears to be protein denaturation and aggregation, which in turn can lead to inhibition of DNA repair, inhibition of *de novo* DNA synthesis, mitotic catastrophe, cytoskeleton damage, and loss of membrane integrity [5, 9, 10]. Another mechanism influencing cell death is HT-induced antitumour immunity, tumour vascular damage resulting in decreased blood flow, and increased generation of reactive oxygen species [5, 7, 10]. In addition to the direct cytotoxic effect, HT is also known to impacts the immune system. There has been compelling evidence that HT can induce abscopal effects mediated by an activated immune response [11]. HT-induced increased expression of T cells (CD3+ and CD8+ cells) is known to evoke abscopal antitumour immune effects [12]. Clearly, hyperthermia’s pleiotropic effects and favorable history substantiate its further investigation into clinical use.

A relatively newer approach that has seen promising advancement as a therapy in oncology is the stimulation of microbubbles by ultrasound. Microbubbles injected intravenously can flow freely through microvasculature because of their stability and small size (3–4μm), and have been traditionally used as contrast agents in diagnostic ultrasound imaging. Studies have indicated that exposing microbubbles to acoustic pressure at or near their resonant frequencies results in bubble oscillations. Two types of bubble oscillations are known to occur namely stable cavitation and transient cavitation also known as the inertial cavitation. Stable cavitation usually occurs at lower ultrasound pressures whereas inertial cavitation occurs at high acoustic pressures. Under higher acoustic pressures, the size of microbubbles increases drastically followed by a violent collapse fragmenting into several tiny bubbles. The cavitation phenomenon of microbubbles can produce bioeffects on neighboring endothelial cells such as increasing the permeability of the plasma membrane [13, 14]. The structural disruption to the plasma membrane has been reported to cause increased vascular permeability, decreased vascular integrity and endothelial cell death depending on the acoustic parameters used [13, 15]. In recent years, ultrasound-stimulated microbubbles (USMB) mediated drug and gene delivery is receiving considerable attention. Cavitation of microbubbles caused by ultrasound pulses leads to transient pore formation at the plasma membranes of cells facilitating the extravasation of drugs and genes [16, 17]. This technique has also been applied for permeabilizing biological barriers such as the brain tumour microvessels/capillaries and or the blood-brain barrier (BBB). USMB-induced disruption of BBB allows the administration of therapeutic agents that can be used to treat various neurological disorders [17–19]. USMB can also be employed to selectively deliver therapeutic agents across the extracellular matrix (ECM). In most solid tumours targeting chemotherapeutics across the ECM remain challenging due to the chaotic network of extracellular macromolecules and increased interstitial fluid pressure (IFP). Ultrasound combined with microbubble can overcome this limitation by lowering the content of IFP and subsequently allowing the penetration of therapeutic drugs deeper into dense ECM. USMB has also been used as drug and gene delivery carriers. Microbubbles used as carriers of therapeutic agents are burst by an ultrasound beam at the targeted area. Drug or gene-loaded ultrasound-targeted microbubbles technique has been utilized successfully for the treatment of various diseases [20–23]. Also, USMB as the carrier transport for proteins and lipids has shown enhanced tumour response in an *in vivo* model [24]. Furthermore, oxygen-carrying microbubbles are used to enhance the effect of various cancer treatment modalities [25]. The use of USMB for delivering oxygen to the hypoxic region in tumour microenvironment has shown exceptional outcomes paving the way to clinical trials. More recently, several groups have evaluated the efficiency and effectiveness of ultrasound and microbubble-mediated drug delivery in three dimensional (3D) tumour spheroids models. The spheroids models are of great clinical significance because they can accurately mimic the complexity of the human solid tumour biology and microenvironment such as the vasculature and ECM. Studies suggest that USMB can enhance the penetration of several drugs and localized the release of drugs into the target tissue of spheroids models. Grainger *et al* demonstrated 6–20 fold increase in penetration of nanoparticles in breast cancer 3D *in vitro* tumour spheroids. The study showed that prolonged ultrasound pulses and increased ultrasound exposure led to greater particle penetration [26]. Additionally, doxorubicin-liposome-loaded USMB has shown to internalized deeper into the layers of the 3D tumour spheroid thereby reducing the number of viable tumour cells [27]. Thus, the implementation of USMB in 3D tumour spheroids holds great promise to improve the delivery of chemotherapeutics agents that may have the potential for successfully treating various clinical diseases in the future. Recent research in radiation oncology has demonstrated that microbubbles mediated membrane-damage can also significantly enhance the effectiveness of radiation therapy [15]. Pre-clinical experiments have shown a synergistic response in tumour cell death and vascular disruption when USMB are administered in combination with radiation therapy [15]. The potentiation of radiation damage has been attributed to endothelial perturbation stimulating activation of specific genetic pathways, which sensitize the tumour to subsequent therapeutic application. This phenomenon is evidenced to work through the ceramide-mediated acid sphingomyelinase (ASMase) signaling pathway leading to endothelial apoptosis [15, 28–30]. Some studies have also examined the combinational effect of USMB and chemotherapy. Exposure to USMB is known to create transient pores on the cell membrane resulting in enhanced cell permeability that elevates the uptake of various drugs used for cancer treatment. Several preclinical studies have confirmed increased tumour response followed a combination of USMB and chemotherapy. One of the mechanisms leading to enhanced tumour response followed these two therapies is known to be a result of the rapid shutdown of tumour blood flow subsequently resulting in secondary cellular events. A study conducted with the PC3 xenograft model demonstrated a significant reduction in tumour perfusion within 24 hours followed by an increase tumour cell death combining docetaxel and USMB [31]. Similar results were reported in breast tumour xenograft when metronomic cyclophosphamide drug was used in combination with USMB [32]. Some report suggests that both USMB and chemotherapy have vascular disrupting properties. Work by Czarnota and group has shown USMB to cause extensive tumour vascular endothelial damage leading to enhanced tumour response. Similarly, *in vitro* studies performed with paclitaxel and docetaxel have shown to inhibit the proliferation of endothelial cells resulting in overall vascular density reduction [33]. Thus, the combination of USMB with radiation and or chemotherapy has shown to be a potential treatment strategy by targeting tumour vasculature. These studies introduce an adjunctive cancer therapy that can be spatially targeted by confining the acoustic fields to the tumour, thereby sparing normal tissues.

Whereas current translational research continues to optimize the USMB radiosensitization effect, the study here aims to investigate whether USMB-mediated tumour response also enhances HT cancer therapy. Combining the spatial targeting of USMB treatment with the tumour selective toxicity of HT treatment creates a novel therapy regimen desirable for sparing healthy tissue. The study here investigates this new combination therapy in a well-vascularized tumour model. Human breast cancer line MDA-MB-231 xenografts were grown in the hind limbs of mice and exposed to USMB treatment alone, HT treatment alone, or a combination of USMB and HT. The parameters of each treatment were modulated in order to optimize the treatment approach, giving rise to a large range of experimental conditions. Animals were first exposed to USMB followed with HT after 5 hours. The selection of a 5 hours time interval between these two treatments was based on our previous finding. Enhanced tumour response was observed when two treatment modalities were separated by more than 5 hours. [15]. Tumours were analyzed 24 hours after treatment using histological assessment to determine levels of tumour cell death and vascular disruption.

## Materials and methods

### Cell tissue culture

MDA-MB-231 breast cancer cells were obtained from the American Type Culture Collections (ATCC, MD, USA), and cultured in RPMI–1640 medium (Wisent BioProducts) supplemented with 10% fetal bovine serum (Sigma-Aldrich) and 1% penicillin/streptomycin antibiotic (ThermoFisher Scientific). The cells were incubated at 37°C in 5% CO2 and once ≥80% confluency was reached cells were trypsinized using 0.05% Trypsin-EDTA (Wisent BioProducts) and split for continuing passage or prepared for injection. For injection, cells were centrifuged and re-suspended in Mg+/Ca+ Dulbecco’s Phosphate Buffered Saline (DPBS) at a concentration of 5 x 10^6^ cells/ 100 μL.

### Animal model

Female SCID-B17 mice were obtained from Charles River Canada. A cell suspension of 100μL containing 5 x 10^6^ cells was injected subcutaneously in the hind limbs using a 27 gauge needle. During injection, mice were anesthetized with 2% isoflurane in an O2 flow of 1.5 L/h to eliminate any pain perception. Xenograft tumours developed in a 4 to 6 week period, and would be exposed to therapy once they had reached 5–10 mm in diameter. Prior to treatment, mice were anesthetized briefly with isoflurane, followed by an intraperitoneal injection mixture of ketamine (100 mg/kg) and xylazine (5 mg/kg) at a volume of approximately 100 μL or adjusted to body weight. Throughout the treatment time, mice were visually monitored and body temperature was regulated with heat lamps and heat pads.

All animal experiments were conducted in compliance with local animal welfare laws, guidelines and policies approved by the Sunnybrook Research Institute Institutional Animal Care and Use Committee.

### Experimental design

**Fig 1:**
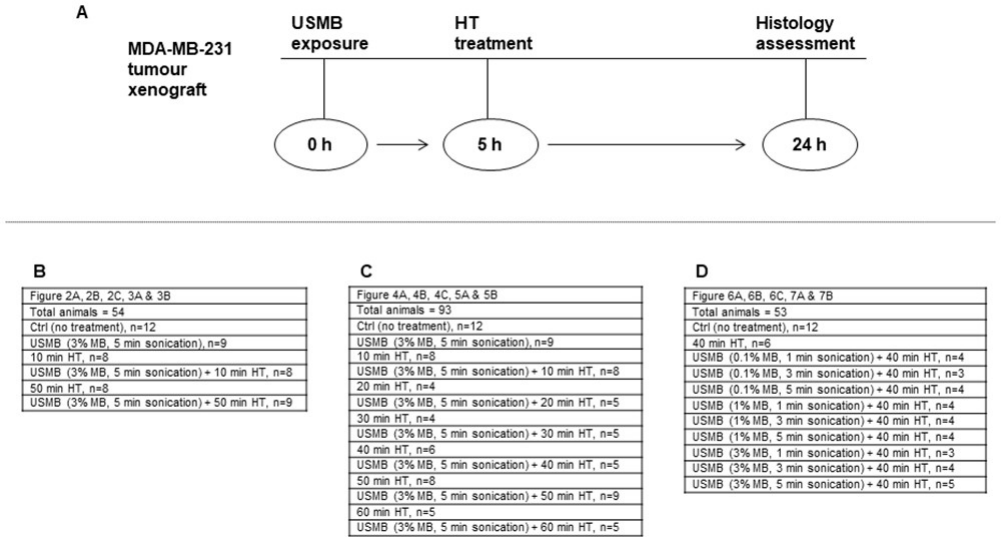
Schematic overview of the experimental design. (A) Diagram depicting experiments carried at each time point. Animals were first exposed to USMB consisting of different concentrations of microbubble (0.1%, 1%, and 3%) and different sonication times (1, 3, and 5 min) at constant peak negative pressure of 570 kPa. Five hours after USMB treatment mice were exposed to 43°C water bath HT for 10, 20, 30, 40, 50, and 60 minutes. Tumour sections were collected for histological assessment at 24 hours followed treatment. Tables (B), (C), and (D) display different parameters incorporated in the study. Ctrl = Control; USMB = ultrasound-stimulated microbubbles; MB = microbubble; HT = hyperthermia; min = minutes; h = hour.

This study aimed to investigate the biophysical parameters of USMB and HT for enhancing tumour response in the breast tumour xenograft model. Different microbubble concentrations, ultrasound exposure time, and HT duration were incorporated in the study. A peak negative pressure of 570 kPa was used for the disruption of microbubbles possibly achieved through inertial cavitation. Mice were first exposed to USMB treatment followed with HT after 5 hours. The work used 200 tumour-bearing mice. To elucidate whether USMB treatments have a similar enhancement-of-treatment effect with HT comparable to radiation therapy, six conditions were investigated; control (no treatment), USMB only (3% (v/v) microbubble concentration, 5 minutes ultrasound exposure), HT alone (10 minutes or 50 minutes), and USMB combined with HT (10 minutes or 50 minutes) for a total of 54 animals. In order to investigate the optimization of HT parameters, six different heating durations were carried out (10, 20, 30, 40, 50, 60 minutes), with and without USMB for a total of 14 conditions with inclusion of control and USMB alone groups (93 animals). In order to optimize USMB parameters, three different microbubble concentrations (0.1%, 1%, 3% v/v) and three different ultrasound exposure durations (1, 3, 5 minutes) were combined with 40 minutes of HT for 9 new parameter combinations and a total of 11 conditions after including control and HT only groups (53 animals). A table displaying all the permutations and number of animals per treatment conditions is included in Fig 1.

### Ultrasound-stimulated microbubble treatments

#### Preparation of microbubbles

Definity^®^ microbubbles, (Lantheus Medical Imaging, Inc., North Billerica, MA, USA) comprised of a liposome shell surrounding perfluoropropane gas, were used in this study. Before treatment, bubbles were warmed to room temperature and subsequently activated using a Vialmix^®^ device (Lantheus Medical Imaging, Inc.) at 3000 rpm for 45 seconds. The microbubble vial was kept inverted to encourage a slight separation of bubble sizes such that bubble size would be relatively uniform when withdrawn from the first few millimeters of the inverted cap. Microbubble concentrations were calculated in accordance to mean mouse blood volume estimated by the animal body weight. Three different concentrations of microbubbles were used in these experiments– 0.1%, 1%, or 3% (v/v), prepared through saline dilutions.

### Ultrasound and microbubble exposure

The ultrasound treatment system consisted of a wave-form generator (AWG520, Tektronix), an amplifier (RPR4000, Ritec), a digital acquisition system (Acqiris DC440/PXI8570, Agilent Technologies Canada, Mississauga, ON, Canada) and a 2.85 cm unfocused planar ultrasound transducer with 500 kHz central frequency (Valpey Fisher Inc., MA, USA). The transducer was focused at 8.5 cm with a focal point -6 dB beam width of 31 mm. Before experiments, the focal point of the ultrasound beam was adjusted using the digital acquisition system. To ensure that the maximal signal was received at the focus, a reference needle was placed in front of the transducer that was monitored through the system. For treatment, mice were anesthetized using ketamine and xylazine and were immersed in a 37°C water bath with only the lower body and limbs submerged, and tail pointed up. The tumour-bearing limb was secured directly in front of the middle of the therapy transducer at a distance calibrated for the maximal focused signal (8 cm). Animals were injected with a 100 μL bolus of the microbubble dilution followed by a 150 μL 0.2% heparin-saline flush through a tail-vein catheter. Immediately after microbubble injection, animals were exposed to ultrasound for 1, 3, or 5 minutes. Tumours were exposed to a 16-cycle tone burst over 50 milliseconds at 500 kHz with a 3 kHz pulse repetition frequency (0.05 seconds total) followed by no ultrasound for 1.95 seconds to permit blood vessels to refill with microbubbles for total pulse sequence duration of 2 seconds. This pulsing sequence was continuously repeated over 1, 3, or 5 minutes exposure duration. All treatments were carried out at a peak negative pressure of approximately 570 kPa which corresponds to a mechanical index of approximately 0.8 at the focus as measured with a calibrated hydrophone [15, 34, 35].

### Hyperthermia treatments

The HT treatments were administered 5 hours after USMB exposure as preliminary work indicated this is an appropriate time for a prominent vascular-related death effect [15]. For the HT treatment, a water bath was heated to a controlled temperature of 43°C. Mice were secured upright in a ventilated tube such that only their tumour-bearing limb was submerged in the water bath. The HT treatment durations investigated included 10, 20, 30, 40, 50, or 60 minutes.

### Histology

Mice were euthanized 24 hours after USMB and HT treatment. Excised tumours were dissected in half and fixed in 10% neutral buffered formalin for 48 hours at room temperature followed by transfer to 70% ethanol to avoid over-fixation. Specimens were then embedded in paraffin blocks, sectioned on glass slides, and stained with hematoxylin and eosin (H&E) for a qualitative look at cell morphology. Tumour specimens were also labeled with terminal deoxynucleotidyl transferase dUTP nick-end labeling (TUNEL), as an assay of cell death, and a cluster of differentiation 31 (CD31) as an assay of the vascular index (Pathology Research Program, University Health Network, Toronto, ON, Canada).

TUNEL detects DNA fragmentation. For the cell death assay, low-magnification images of tumour slides were acquired with a light microscope (Leica MZ FL III, Leica Microsystems, Concord, ON, Canada), and digitized for analysis. The percentage of cell death was quantified from digitized TUNEL files using an in-house custom code developed in MATLAB. The percentage of cell death per tumour per experimental condition was averaged.

CD31 immunostaining targets a protein concentrated in the intercellular junctions of endothelial cells. For the vascular index assay, 10–20 high-magnification (20x objective lens) images of tumour slides were acquired with a light microscope in a consistent pattern for each tumour (periphery and centre). The number of intact blood vessels were manually counted using ImageJ (NIH, Bethesda, MD, USA) software and averaged per sample. The vascular index was deemed as the mean number of viable blood vessels per tumour per experimental condition.

### Statistical analysis

Statistical tests consisted of one-way ANOVA and two-way ANOVA where applicable to determine significant trends and factors. To determine statistical significance between groups, a t-test with Welch’s correction was performed. All tests were carried out using Graph Pad Prism software version 6 (Graph Pad Software, La Jolla, CA, USA).

## Results

The aim of this study was two-fold: to investigate the potentiation ability of USMB treatment in an HT therapy regimen, and to optimize the combined treatment parameters in an MDA-MB-231 breast cancer xenograft model.

### USMB combined with HT results in increased cell death and vascular disruption in MDA-MB-231 xenografts

**Fig 2:**
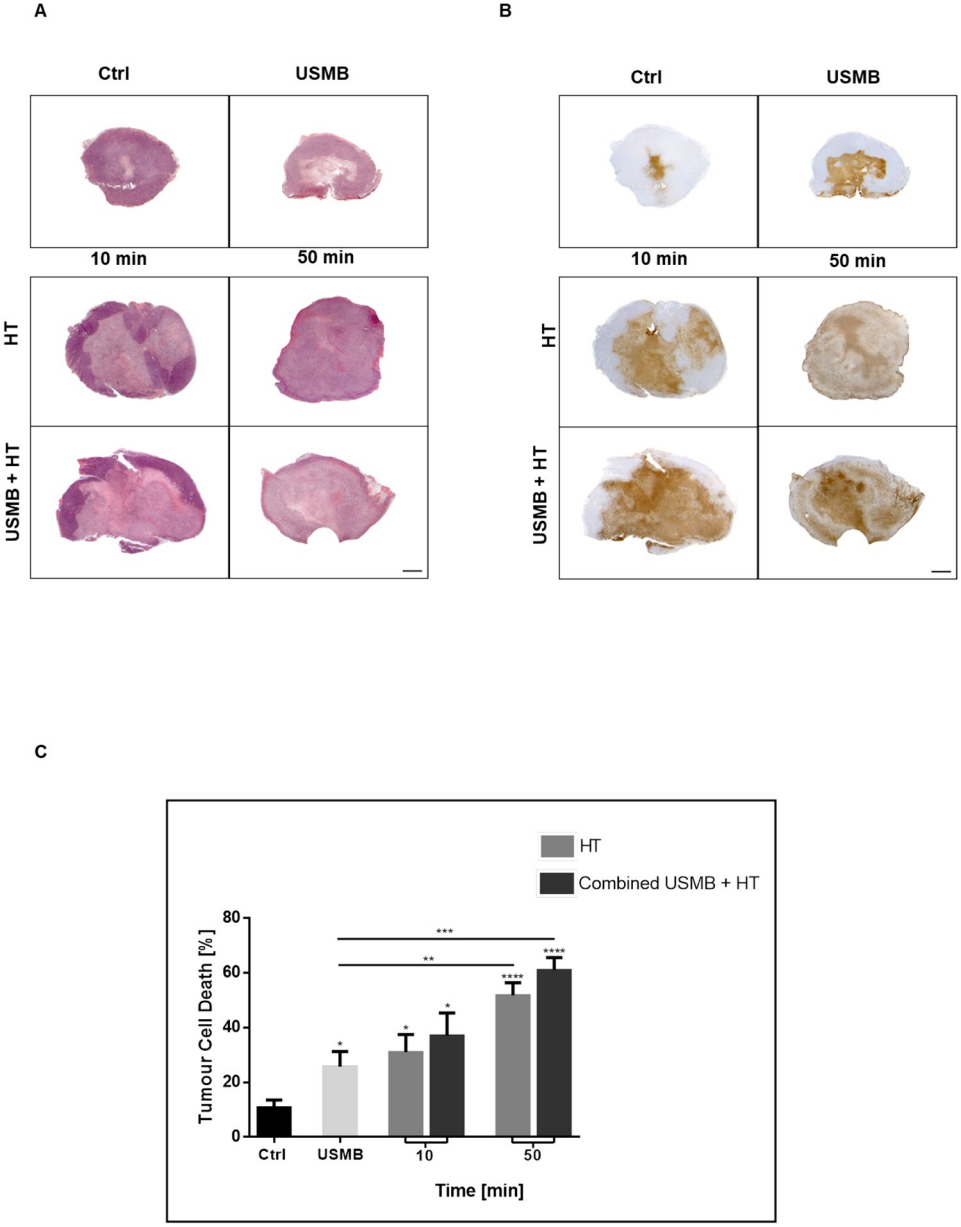
Gross tumour histological evaluation and quantification of cell death. Low magnification light microscopy images of representative MDA-MB-231 xenograft sections stained with (A) H&E and (B) TUNEL. Groups were treated with either nothing (control), USMB alone, HT at low (10 minutes) or high (50 minutes) heat durations, or a combination of both USMB and HT. All treatments conferred varying amounts of detectable cell death (brown TUNEL stain). The scale bar denotes 1mm. (C) The cell death assay was determined from TUNEL stained tumour sections (Fig 2B) and is expressed as a percentage of the total tumour area. As treatment parameters were increased in the intensity of anticipated cytotoxicity, there was an observed step-wise increase in cell death. Statistical significance, determined by Welch’s corrected t-test, is indicated for each treatment condition compared to control with P values of 0.01–0.05 (*), 0.001–0.01 (**), 0.0001–0.001 (***), and <0.0001 (****). Error bars indicate standard error of the mean. A total of 54 animals were included in this data set. Ctrl = Control; USMB = ultrasound-stimulated microbubbles; HT = hyperthermia; min = minutes.

Results indicated that USMB alone, HT alone, and a combination of USMB/HT were effective treatments for cell death in MDA-MB-231 xenografts. Histological evaluation of H&E (Fig 2A) and TUNEL (Fig 2B) stained tumour sections were used to assess cell death. Large areas of cell death, determined from coincident TUNEL and H&E stained sections, were found in all treated tumours compared to controls. In areas negative for dark brown TUNEL stain, the corresponding H&E regions appeared stained a dark purple due to the blue-purple color of hematoxylin stained nucleic acids. In areas positive for TUNEL, the absence of a homogenous hematoxylin nuclei stain, reminiscent of nuclear fragmentation in cell death, left a paler pink pattern (Fig 2A&2B) consistent with cellular necrosis. Statistical analysis (ANOVA) of the quantified TUNEL assay indicated a significant difference between groups (p<0.001) (Fig 2C). The untreated controls indicated a central area of baseline tumour cell death which, when quantified, represented 11 ± 3% of the total tumour area. Exposure to USMB alone caused a significant increase in cell death compared to controls with an affected area of 26 ± 5% (p<0.05). The heat only treatments of 10 minutes and 50 minutes at 43°C resulted in 31 ± 6% and 52 ± 5% cell death respectively, with increasing significance when compared to control (p<0.05 and p<0.0001). Adding USMB to both HT groups increased the cell death response to 37 ± 8% and 61 ±5%, respectively. Only the 50 minute HT groups, with and without USMB, demonstrated a significant increase in cell death from the USMB alone treatment. To uncover optimal heat duration, more HT groups were added and results are described below.

**Fig 3:**
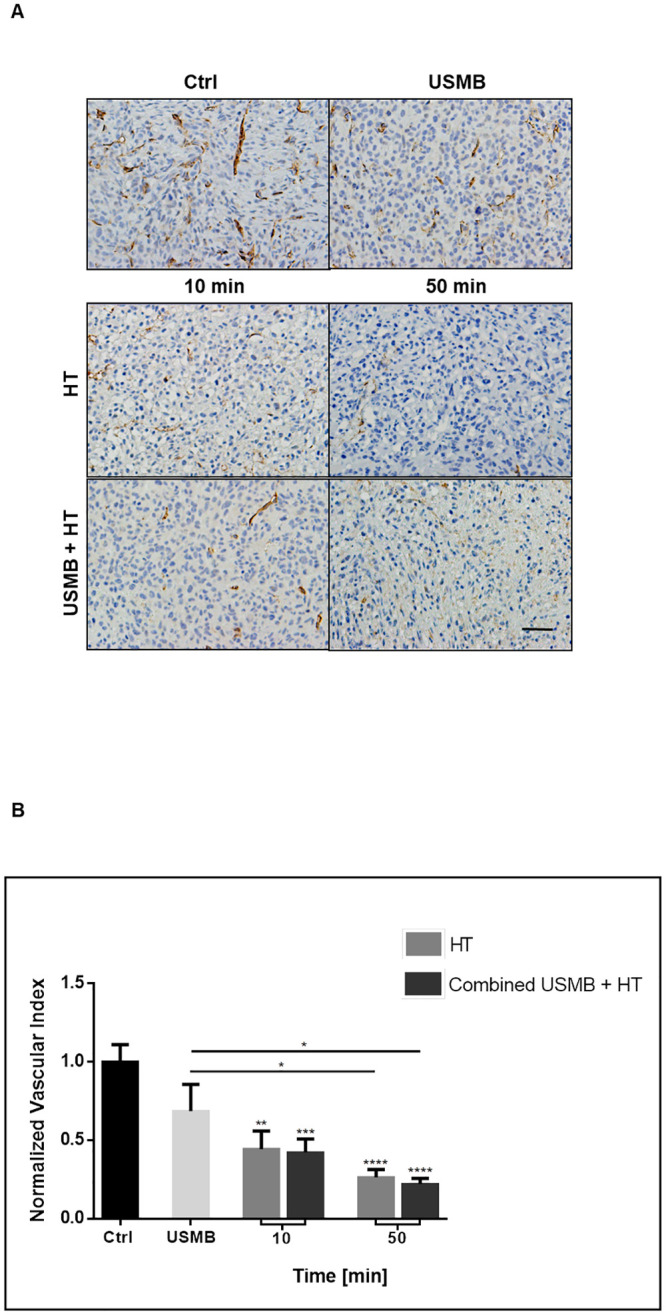
Histological evaluation of vasculature and quantification of the vascular index. (A) High magnification light microscopy images of CD31 stained tumour sections. Groups were treated with either nothing (control), USMB alone, HT at low (10 minutes) or high (50 minutes) heat durations, or a combination of both USMB and HT. Representative images for each treatment condition show intact blood vessels as morphologically well-defined and stained brown. Control samples showed a high density of intact blood vessels, while treated groups had decreased numbers. Images were taken at a 20x objective lens, and the scale bar denotes ~50μm. (B) The vascular index was determined from CD31 stained tumour sections and represents a mean number of intact blood vessels in tumour sections. The step-wise trend for decreasing vascular index mirrors the increase in cell death as shown in Fig 2C. Statistical significance, determined by Welch’s corrected t-test, is indicated for each treatment condition compared to control with P values of 0.01–0.05 (*), 0.001–0.01 (**), 0.0001–0.001 (***), and <0.0001 (****). Error bars indicate standard error of the mean. A total of 54 animals were included in this data set. Ctrl = Control; USMB = ultrasound-stimulated microbubbles; HT = hyperthermia; min = minutes.

Changes in the vascular index were evaluated using CD31 immunohistochemistry. High magnification images of tumour sections revealed intact blood vessels as distinct, dark brown stained endothelial cells (Fig 3A). It was noted that the intact vessels, positive for CD31, were predominantly observed in the periphery of the treated tumours where there was an absence of TUNEL stain in the corresponding slides. The vascular index, representing the average number of intact vessels, was significantly lower compared to corresponding samples in all groups treated with HT. The 10 minutes and 50 minutes HT treatments resulted in decreases to 0.4 and 0.3 initial vascular indexes, respectively, compared to control (Fig 3B). The addition of USMB to HT resulted in a further decrease in the vascular index in both cohorts. Similar to the cell death assay, only the 50 minutes HT groups had a significantly reduced vascular index when compared to the USMB only treatment. Taken together with the TUNEL assay data, these results indicate that combining USMB with HT causes an increase in cell death in tumours, which coincides with a decrease in vascular index.

### Hyperthermia treatment dose curve plateaus early

**Fig 4:**
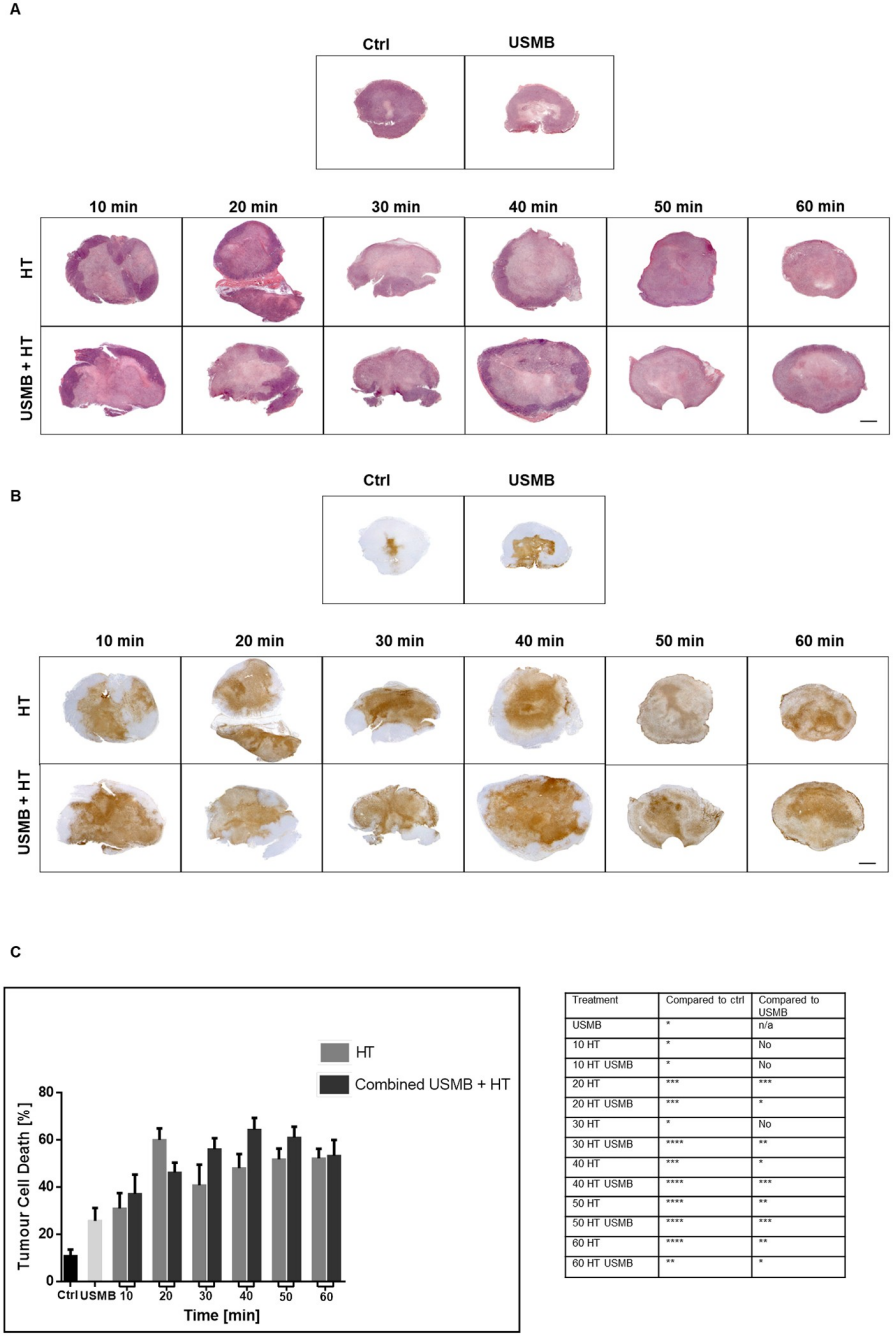
Analysis of tumour cell death response with the addition of more HT groups. Low magnification light microscopy images of tumour sections with added heat groups to encompass 10, 20, 30, 40, 50, and 60 minutes HT treatment durations and the combined treatment (USMB and HT) complements. Also shown are representative images for control and USMB only groups. (A) Tumour sections stained with H&E and their corresponding TUNEL stained sections (B). All treatment groups including HT (alone or in combination with USMB) appeared to have large areas of cell death. The scale bar denotes 1mm. (C) Quantification of tumour cell death revealed a plateau in the cytotoxicity response to HT treatment occurring as early as 10 minutes at 43°C. Statistical significance, determined by Welch’s corrected t-test, is indicated for each treatment condition compared to control with P values of 0.01–0.05 (*), 0.001–0.01 (**), 0.0001–0.001 (***), and <0.0001 (****). Error bars indicate standard error of the mean. A total of 93 animals were included in this data set. Ctrl = Control; USMB = ultrasound-stimulated microbubbles; HT = hyperthermia; min = minutes; n/a = not applicable.

In order to explore the dose-response relationship of MDA-MB-231 xenografts to the HT treatment, further heat-experimental groups were added to include 20, 30, 40, and 60 minutes duration at 43°C alone or in combination with USMB. Histological assessment of H&E and TUNEL (Fig 4A&4B) revealed large areas of cell death in all treated groups, with a tendency for affected areas to be central and relatively unaffected areas (if any) to be peripheral. Quantifying the tumour response to increasing durations of HT revealed an initial rise in cell death at the shorter durations followed by a plateau with little added treatment effect in response to longer durations (Fig 4C). The step-wise increasing cell death trend seen earlier in Fig 2C was less definite with the addition of additional heating times. All groups, HT alone, USMB and HT combined, resulted in significant increases in cell death when compared to control. The addition of USMB to the HT treatments consistently increased cell death in all groups except for the 20 minutes HT group. The largest increase in cell death from a baseline of 11 ± 3% in controls to 64 ± 5% was observed with the application of USMB combined with 40 minutes of HT (Fig 4C). When statistically comparing HT groups to the USMB only cohort, HT duration of 40 minutes or longer produced consistently more cell death in both HT alone and when combined with USMB. Therefore, further experimentation involved 40 minutes of heat as the HT regimen.

**Fig 5:**
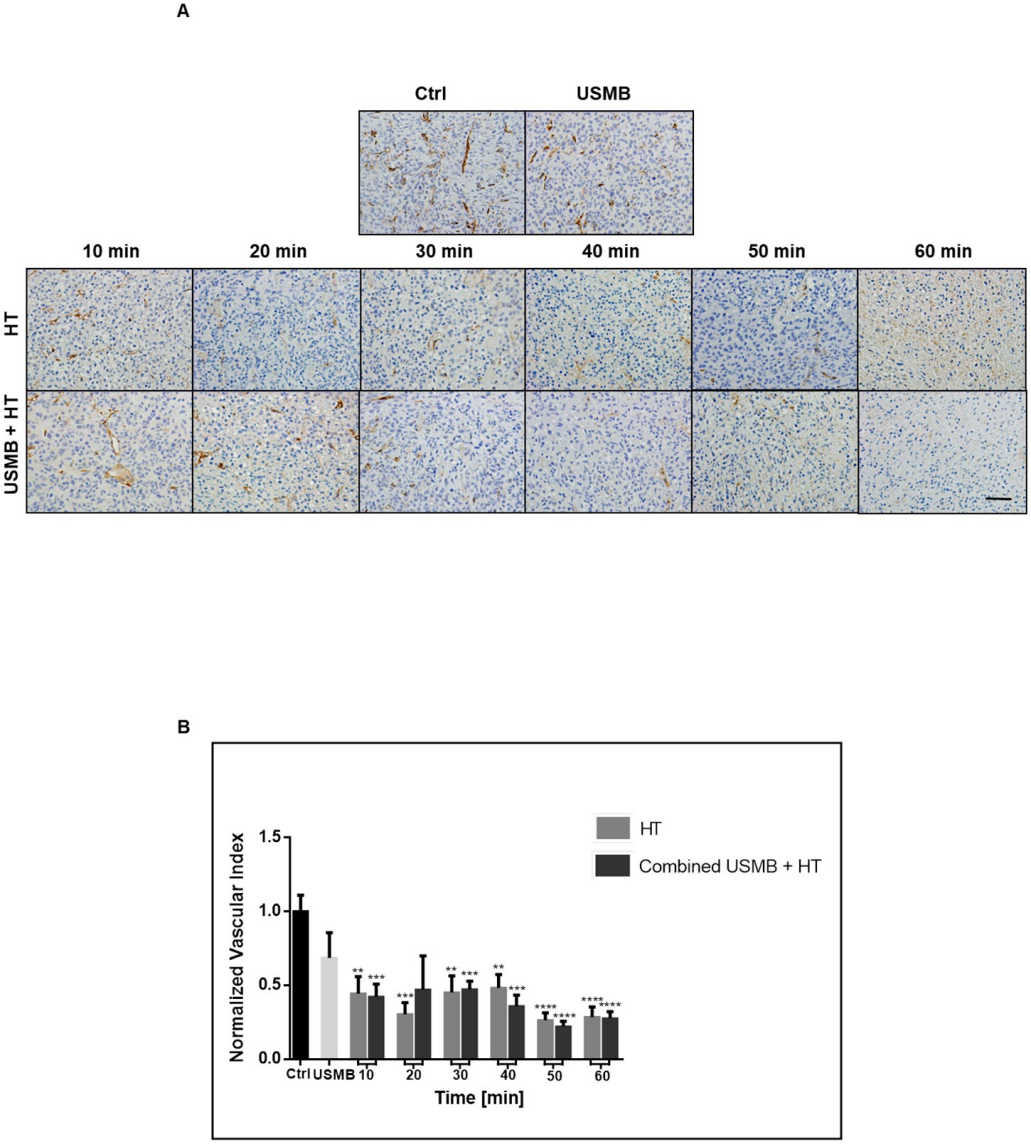
Analysis of tumour vasculature with the addition of more HT groups. (A) High magnification images of tumour sections stained for CD31. The vasculature becomes less evident in the treated groups, with decreasing numbers of stained blood vessels. Images were taken at a 20x objective lens, and the scale bar denotes ~50μm. (B) Calculation of the vascular index from CD31 analysis revealed a similar reduction in intact blood vessels in all groups exposed to HT or USMB and HT. Statistical significance, determined by Welch’s corrected t-test, is indicated for each treatment condition compared to control with P values of 0.01–0.05 (*), 0.001–0.01 (**), 0.0001–0.001 (***), and <0.0001 (****). Error bars indicate standard error of the mean. A total of 93 animals were included in this data set. Ctrl = Control; USMB = ultrasound-stimulated microbubbles; HT = hyperthermia; min = minutes.

The vasculature analysis displayed an inversed but similar trend to the cell death analysis, with a rapid decrease and plateau of the vascular index as HT duration increased (Fig 5B). High magnification images of CD31 labeled tumour sections exhibited prominent blood vessels throughout the untreated control samples; however, blood microvessels appeared to be fewer in number and less obvious in treated groups (Fig 5A). Statistical analysis using ANOVA revealed a significant difference in the observed trend, with Welch’s t-test comparison indicating all HT and USMB combined with HT groups having a significantly lower vascular index when compared to controls, with one exception (the 20 minutes combined group).

### Increase in cell death at low USMB doses: HT treatment effect

**Fig 6:**
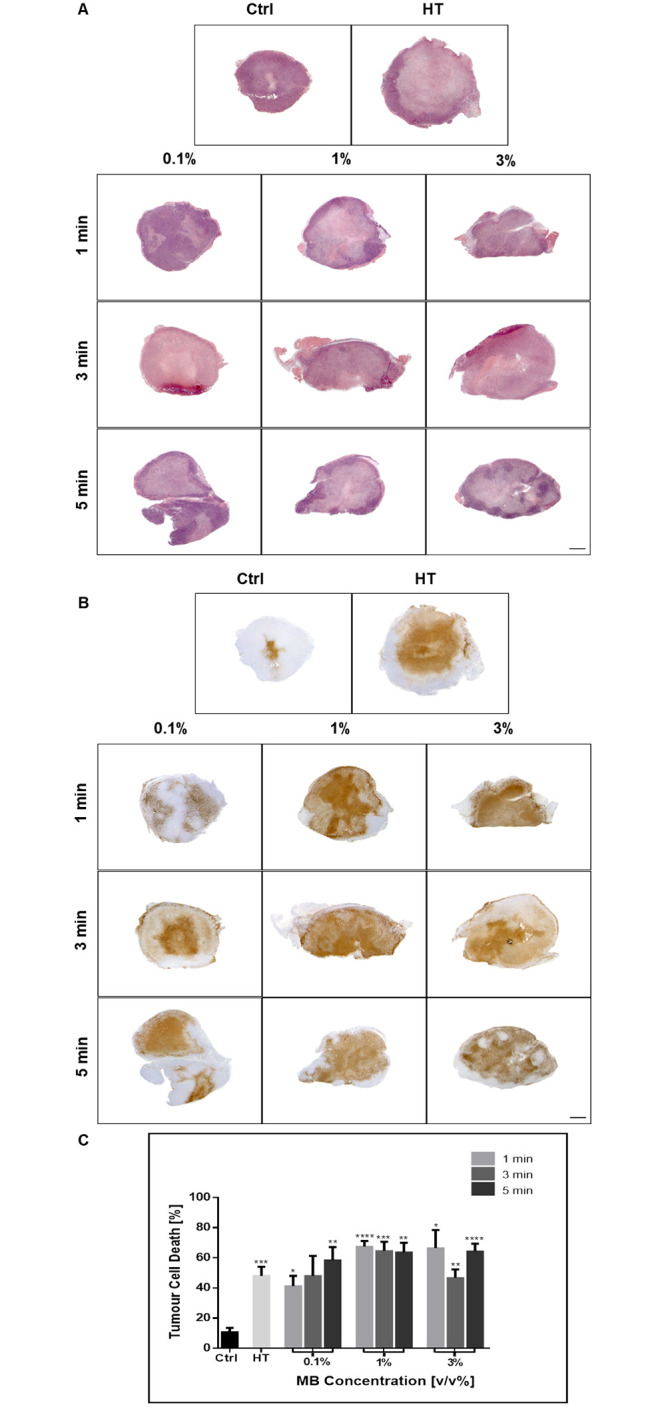
Analysis of tumour cell death response with the addition of more USMB groups. Low magnification light microscopy images of tumour sections with varying microbubble and ultrasound therapy parameters. (A) Representative H&E stained tumour sections and corresponding (B) TUNEL stained sections. Treated groups showed large amounts of cell death compared to controls. (C) Cell death was quantified from TUNEL analysis and indicated that low, medium and high USMB parameters all result in similar amounts of cell death. Statistical significance, determined by Welch’s corrected t-test, is indicated for each treatment condition compared to control with P values of 0.01–0.05 (*), 0.001–0.01 (**), 0.0001–0.001 (***), and <0.0001 (****). Error bars indicate standard error of the mean. A total of 53 animals were included in this data set. Ctrl = Control; MB = microbubble; HT = hyperthermia; min = minutes.

**Fig 7:**
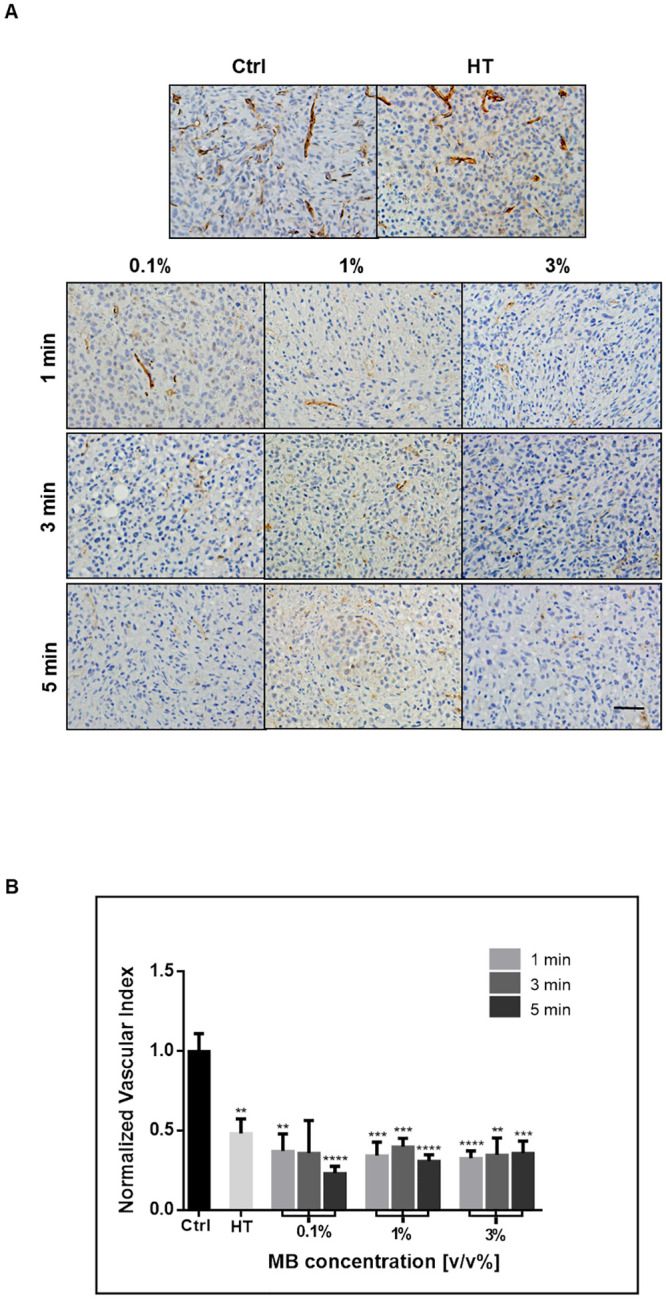
Analysis of tumour vasculature with the addition of more USMB groups. (A) High magnification images of representative tumour sections stained for CD31. The vasculature becomes less evident in the treated groups, with decreasing numbers of stained blood vessels. Images were taken at a 20x objective lens, and the scale bar denotes ~50μm. (B) Vascular index quantified from the analysis of CD31. All treated groups showed a similar decrease in the vascular index compared to untreated controls. Statistical significance, determined by Welch’s corrected t-test, is indicated for each treatment condition compared to control with P values of 0.01–0.05 (*), 0.001–0.01 (**), 0.0001–0.001 (***), and <0.0001 (****). Error bars indicate standard error of the mean. A total of 53 animals were included in this data set. Ctrl = Control; MB = microbubble; HT = hyperthermia; min = minutes.

In order to guide treatment implementation of the USMB, different ultrasound exposure, and microbubble dosing conditions were also carried out. The 3% microbubble concentration and 5-minutes ultrasound exposure parameters used in the above experiments were based on the “high” USMB values producing effective results in earlier work [15]. In order to elucidate the optimal USMB treatment parameters, lower concentrations of 1% and 0.1% was combined with lower sonification times of 3 minutes and 1 minute to include combinations of three microbubble concentrations and three ultrasound exposure times. The qualitative and quantitative cell death assessment revealed that at 40 minutes of HT, both low and high USMB doses result in similar increases in cell death compared to controls (Fig 6A&6B). TUNEL quantification indicated a significant increase in cell death from a baseline of approximately 4 to 6 times with USMB/HT treatment (Fig 6C). Analysis by two-way ANOVA indicated microbubble concentration as a significant factor influencing cell death (p = 0.0011). Further analysis revealed that parameters of 1% microbubble concentration and 1-minute ultrasound exposure resulted in a significant increase in treatment effect compared to 40 minutes of heat alone (p = 0.0254). A corresponding decrease in the vascular index in all treatment groups once again indicated an effective assault on the vasculature following USMB and HT (Fig 7A&7B). While most treated groups were different from control, negligible differences in vascular index existed between treatment groups when compared to each other. Therefore, the efficacy of USMB and HT to cause cell death and vascular disruption can be achieved even at low doses of USMB therapy with an optimal effect observed when 1% microbubble concentration and 1-minute sonification exposure is combined with 40 minutes of HT.

## Discussion

This study investigated the use of USMB in combination with HT as a therapy to treat breast cancer xenografts *in vivo*. Histological evaluation was used to determine treatment effects on cell death and vascular changes. The results from this study demonstrate that USMB and HT when combined act as a potent modality in causing tumour and vascular cell death. Previous studies have investigated the USMB enhancement effect upon radiation therapy *in vitro* and *in vivo* in multiple cell lines [15, 28, 36–38]. Czarnota *et al*. demonstrated that USMB treatment followed by subsequent radiation exposure (2 Gy or 8 Gy) resulted in a synergistic cell death effect in prostate cancer mouse xenografts. The combined treatments demonstrated a supra-additive effect compared to single treatments alone reaching 40–60% cell death measured 24 hours after exposure. A similar synergistic enhancement of radiation therapy was observed in bladder [37] and breast [38] xenograft experiments when USMB treatment was used in combination with radiotherapy. These studies demonstrated the mechanism of action for this synergy to be microbubble-induced ceramide-facilitated endothelial cell death. Stimulating the circulating microbubbles with ultrasound causes the endothelial cells to be perturbed through biomechanical effects such as the activation of pro-apoptotic genetic pathways. An observed increase in ceramide in these experiments was attributed to the activation of pro-apoptotic pathways associated with membrane damage [39, 40]. The resultant endothelial cell death and vascular disruption reduced blood flow to the tumour area as well as caused increased sensitivity to subsequent radiation exposure.

The study here investigated whether a similar enhancement-of-treatment phenomenon would occur when combining USMB with HT instead of radiation. Work done by [41] examining USMB and HT therapy *in vitro* in the mouse mammary tumour cell line 4T1 revealed that the combination of the two therapies synergistically enhanced cell kill. However, the findings displayed here *in vivo* in MDA-MB-231 xenografts indicate that USMB and HT alone or together are an efficacious therapy but do not act synergistically. Since USMB is targeting endothelial cell disruption in the tumour microvasculature, a physiological dependency, and therefore differential response *in vitro* to *in vivo*, is not unexpected. Exposure to treatments of USMB alone, 10 or 50 minutes of HT at 43°C alone, or in combination with USMB all resulted in significant increases in cell death compared to control samples (Fig 2C). Evaluation of tumour vasculature indicated significant decreases in vascular index coinciding with the increases in cell death in treated groups compared to control samples (Fig 3B). Whereas the addition of USMB to the HT groups did indicate an increased treatment effect in almost every case, but not all were significant. This further exemplifies the link between vascular disruption and tumour cell death. MDA-MB-231 xenografts, being a highly vascularized tumour type, may be particularly sensitive to the HT treatments, since the vasculature is markedly affected when HT is given as a monotherapy or in combination with USMB. As well, the tumour cells themselves may be particularly sensitive to heat treatment. Thompson *et al*. conducted a cell sensitivity test to HT in three different breast cancer cell lines *in vitro* (MCF7, MDA-MB-231, and MCF 10A), and found that compared to the other two cancer types, MDA-MB-231 had the least resistance to heat exposure [42]. As human breast cancer types are highly heterogeneous genetically and phenotypically, the functional response to treatments will also be heterogeneous.

The lack of synergism between the two treatment modalities here appears to come from the potency of the HT treatment itself. Comparison with the work from Lai *et al*. in which MDA-MB-231 xenografts were similarly grown in mice and exposed to USMB and radiation, demonstrates the reproducibility of the USMB single treatment effect in the same cell line. The study reported that control tumours displayed a baseline of 10 ± 2% cell death and treatment with USMB resulted in an increased to 26 ± 5% [43]. In the study here, controls and USMB only treated tumours presented with 11 ± 3% and a 26 ± 5% cell death effect, respectively. However, the combination groups in the radiation experiments saw a 3.4-fold and a 2.3-fold increase in effect when USMB combined with 2 Gy and 8 Gy respectively, while the combination groups in the HT experiments saw only a 1.2 fold increase in effect in both 10 and 50 minutes HT treatments. The HT treatment itself has the ability to produce substantial tumour cell death and vascular disruption, and thus the addition of USMB to HT treatment increases the effect albeit not in a synergistic manner.

In the experiments here, exposure to HT was modulated in order to elucidate an optimal therapy regimen. It was observed that as hyperthermic exposure increased in duration in intervals from 10 minutes to 60 minutes, a plateau in treatment effect was reached occurring even at short heating duration (Figs 4 and 5). The thermal dose is defined from the temperature and exposure time to that temperature [44], and at 10 minutes and 43°C, the thermal dose was sufficient to cause significant cell death in MDA-MB-231 tumours. A consequence of different cell lines having variable sensitivities to HT means there will exist thresholds for the thermal dose necessary to trigger cell death unique to each tumour type [45], and the thermal dose has been shown to vary by a factor of 10 between different cell lines [44]. Suggestions for future work include exposure to HT in less than 10 minutes duration to see if the same level of cell death and vascular disruption could be achieved in MDA-MB-231 tumours at lower exposure durations.

Similar to the HT modulation, USMB treatment parameters were modulated in order to determine an optimal treatment combination of microbubble concentration (0.1%, 1%, or 3% v/v) and ultrasound exposure duration (1, 3, or 5 minutes). Assessment of tumour cell death and vascular changes indicated that comparable results could be attained at both low and high microbubble dose combinations (Figs 6 and 7). An interpretation of this data is that the desired treatment effect is already saturated at the lower concentrations of microbubble and sonification times. In accordance with this suggestion, [36] modulated the microbubble concentration and acoustic pressure parameters in combination with radiation doses, and found that at sufficiently high pressure, such as 570 kPa used here, modulating the microbubble concentration had no effect. A similar trend was observed by Kim *et al*. [36] and suggests a saturation effect resulting from higher pressures causing all bubbles to collapse. Therefore, the destruction effect is saturated even at doses lower than the clinically recommended dose for contrast imaging (8 uL/kg used for 0.01% v/v compared to the clinical imaging dose of 10 uL/kg). Although lower doses did not differ from higher doses in terms of cytotoxicity, it was observed that the least variability within groups existed when 1% microbubbles and 1-minute sonification was implemented, producing an increase in cell death when combined with 40 minutes heat significantly more than 40 minutes of heat alone (p = 0.0254). Given this observation, the optimal USMB dose was, therefore, found for these conditions. However, if lower doses of HT were to be investigated, the USMB parameters should be adjusted around the lowest dose of HT that achieves a relevant therapeutic value.

The strength of the HT treatment and the observed lack of ability for USMB to greatly enhance its effect can be understood in terms of treatment mechanism. The purpose of stimulating the microbubbles with ultrasound is to mechanically disturb the tumour blood vessels, thereby activating ceramide mediated stress response pathway leading to endothelial cell death and consequent tumour vascular destruction [15]. Ceramide-dependent cell death signaling mechanisms are triggered by the activation and translocation of ASMase enzyme in the endothelial compartment [46, 47]. ASMase generated ceramide further stimulates the activation of caspases finally leading to cell death [48, 49]. On the other hand, the mechanisms of heat-induced cell death are plentiful and not well understood, however, many studies have determined that aside from its tumour cytotoxic effects, HT can affect tumour blood flow and vasculature [44, 50]. HT treatments with exposure to temperatures 42°C and above are found to cause extensive damage to the tumour vascular bed and alter blood flow in multiple tumour types [44] [50]. HT in the range of 40°C to 45°C is known to induce thermotolerance and induction of heat shock proteins (HSPs) [51–53]. Cells acquiring the state of thermotolerance have shown to correlate with a higher level of HSPs expression. HT-induced protein denaturation/aggregation which subsequently leads to cell death is hindered by the upregulation of HSPs particularly HSP70. Also, HSP70 overexpressing cells are protected from HT-induced ceramide generation and apoptosis. HSP70 inhibits the activity of caspase-3 and Jun N-terminal kinase (JNK)-pathway that are key regulators of ceramide-induced cell death. Studies have confirmed that elevating the level of ceramide can downregulate or disrupt HSP70 expression resulting in apoptosis [54]. At present, it remains unclear as to what mechanisms underlie the enhancement of cell death and changes in vascular index observed in our study following USMB and HT. As 43°C was used in the work here, perhaps vascular destruction is the primary target of both USMB and HT, and ultimately tumour cell death is being modulated by a shared mechanism. The slight increase in cell death observed in the combined USMB and HT combination groups may be due to the first pass attack on vasculature creating tumour regions of low pH, hypoxia, and nutrient deficiency, factors which are known to increase sensitivity to thermal damage [50].

In summary, combining USMB exposure with HT treatment was demonstrated to increase cell death and reduce vascular index in human breast (MDA-MB-231) tumour xenografts when compared to HT alone. The effects of the HT treatment were maximized at 40 minutes of exposure. Optimal effects of combining the 40 minutes HT treatment were observed with a relatively low microbubble concentration of 1% v/v and a short ultrasound exposure time of 1 minute. Whereas this parameter combination provided optimal additive results, the interaction between the two treatments suggests that further optimization can be achieved by reducing the HT dose to increase the contribution of the USMB treatment, further sparing healthy tissue.

Our study here provides an insight into how tumours behave *in vivo* followed a treatment of USMB and HT. The clinical application of this technique remains questionable, however, the use of this combined modality can surely be confined to the targeted tumour volume sparing nearby healthy tissue. USMB treatment can perturb the endothelial lining of the vasculature and might pre-sensitize vascularized regions of the tumour to HT therapy. HT on the other side can treat hypoxic areas within the tumour which otherwise might be difficult to treat with USMB. Thus, the strengths of one treatment modality can overcome the weakness of others. Recently, high intensity focused ultrasound (HIFU), an adaptation of conventional HT has gained rapid clinical acceptance. A focused beam of ultrasound energy is deposited in the targeted area resulting in thermal ablation. HIFU in combination with microbubble has demonstrated to enhance the heating effects [55]. At present, several clinical trials are ongoing combining HIFU and microbubbles. By using this combination, the use of heat and temperature to ablate tissues can be minimized. Thus, HIFU with microbubbles has shown to be a successful treatment strategy for the treatment of various experimental tumours and hence it might play a significant role in future clinical practice.

## Limitations and future implications

The results presented here add to the body of work demonstrating the utility of vascular endothelial disruption mediated by USMB to enhance various cancer treatment modalities. The results revealed greater tumour cell death and reduced vascular index with the combination of USMB and HT. Although promising outcomes were observed within 24 hours, this does not necessarily mean that these techniques will have effective therapeutic responses. Further studies are needed to assess the long-term effectiveness of this combined treatment. There are also limitations associated with this study that are worth noting. Firstly, the temperature uniformity in tumours is a critical aspect for the activation of several cell death-related signaling pathways. Therefore, incorporating technology that allows temperature mapping and homogenous energy distribution in tumours may be beneficial. Secondly, when using HT as a multimodality treatment, treatment sequencing and the time interval between the two treatments can impact tumour response eminently. Therefore future studies focusing on the sequence, and the time interval with HT treatment might help to understand the influence of these factors on tumours in a better way. Other studies have pointed to the importance of ceramide in microbubble-induced effects and heat shock proteins in HT work but these were not studied here. Furthermore, the translation of these research findings to the clinical settings will require the optimization of these treatment combinations in larger tumours and or orthotropic tumour models.
